# Effectiveness of an online vaccine misinformation game for Arabic-speaking Australians: a randomized controlled trial

**DOI:** 10.3389/fpubh.2026.1768612

**Published:** 2026-05-28

**Authors:** Sophie Vasiliadis, John Cook, Kifarkis Nissan, Suzanna Vidmar, Anneke C. Grobler, Wendy Cook, Kate Hopkins, Chelsey Lepage, Angus Thomson, Margie Danchin, Jessica Kaufman

**Affiliations:** 1 Murdoch Children’s Research Institute, Melbourne, VIC, Australia; 2 Melbourne School of Psychological Sciences, The University of Melbourne, Melbourne, VIC, Australia; 3 Foundation House, Melbourne, VIC, Australia; 4 Department of Paediatrics, The University of Melbourne, Melbourne, VIC, Australia; 5 Wendy Cook Design, Melbourne, VIC, Australia; 6 Sabin Vaccine Institute, Washington, DC, United States; 7 Irimi Company, Lyon, France; 8 The Royal Children’s Hospital Melbourne, Melbourne, VIC, Australia

**Keywords:** gamified information presentation, misinformation, psychological inoculation, randomized controlled trial, vaccines

## Abstract

**Background:**

Vaccine misinformation can undermine vaccine confidence, institutional trust, and uptake. Arabic speakers in Australia are disproportionately susceptible to vaccine misinformation and have experienced adverse health outcomes. This randomized controlled trial tested the effectiveness of *Cranky Uncle – Vaccine (Arabic)*, a gamified psychological inoculation intervention, in improving vaccine attitudes, misinformation discernment, and institutional trust among Arabic-speaking Australians.

**Methods:**

Participants (*N =* 198) were randomized 1:1 to either the intervention (game) or control (educational climate change video). Outcomes were assessed immediately and 3 weeks post-activity using a combination of validated and custom instruments: (1) the Vaccine Trust Indicator (VTI); (2) a misinformation discernment scale developed by the research team, and (3) items from the Institutional Trust subscale. Analyses were conducted using multiple imputation and adjusted linear regression models. The trial protocol was registered in the Australian and New Zealand Clinical Trials Registry (registration number: ACTRN12624000934549p).

**Results:**

No statistically significant differences were found between the intervention and control groups at either timepoint across primary or secondary outcomes, and no subgroup effects were observed by age or education. *Post-hoc* item analyses found that items assessing trust in scientists and trust in pharmaceutical companies demonstrated the greatest difference between groups at the immediate post-activity timepoint.

**Discussion:**

This trial is the first RCT of the *Cranky Uncle – Vaccine* game. Findings highlight methodological and measurement challenges in misinformation research. Developing and validating standardized measures that are appropriately sensitive to detect meaningful changes should be an urgent priority for the field. Future evaluation of the *Cranky Uncle* intervention for different populations could consider testing delivery in structured educational group settings.

## 1 Introduction

Vaccines have long been the subject of misinformation (1), but the COVID-19 pandemic escalated misinformation’s spread and impact (2, 3). A systematic review comparing 33 studies from Europe, North America and Asia found that approximately three in four people believe in at least one conspiracy theory, and this prevalence increased over time (4). In 2020, an estimated 66% of Australian adults believed at least one piece of COVID-19 misinformation, and 14–32% held a vaccine-related conspiracy belief (5). Growing evidence shows that exposure to vaccine misinformation is strongly associated with higher levels of vaccine hesitancy and lower levels of vaccine confidence and institutional trust, as well as reduced vaccine uptake (6–11).

Arabic-speaking populations in the Australian state of Victoria experienced higher morbidity and mortality from COVID-19 and lower rates of COVID-19 vaccine uptake compared to other ethnicities between 2020 to 2022 (12). One factor contributing to this was the widespread circulation of vaccine misinformation in this community (13, 14), which was amplified throughout the pandemic and continues to persist (14, 15).

Migrant communities are particularly vulnerable to misinformation as they are often poorly reached by mainstream English language media and regularly access information from overseas, including their country of origin (16–18). They may also have lower levels of digital fluency, health literacy, and institutional trust (19), which may be caused by socioeconomic disadvantage (20, 21), social exclusion (22), under-representation and historical mistrust from unethical treatment in health research (23, 24). However, there are currently few interventions to address vaccine misinformation that can be tailored to these populations (18).

Emerging evidence suggests that it is possible to psychologically ‘inoculate’ people against the misinformation belief (25, 26) through controlled exposure to real-world misinformation and explanation of how to identify it (27, 28). The COVID-19 pandemic inspired several psychological inoculation interventions for health misinformation (29) that use games which can be shared through social media and feature levels, points and a progress bar to engage users (30). There is growing evidence indicating their effectiveness in inoculating against health misinformation (31, 32). For example, a randomized controlled trial found that gamified inoculation reduced perceived credibility of misinformation significantly more than graphic inoculation (33).

One example of gamified inoculation is the *Cranky Uncle – Vaccine* game (34), a free smartphone and browser game that leverages the principles of psychological inoculation. In this randomized controlled trial, we aimed to assess the effectiveness of the *Cranky Uncle – Vaccine (Arabic)* game in improving vaccine attitudes, teaching players to recognize vaccine misinformation, and increasing institutional trust, immediately and 3 weeks after playing the game, compared with the control condition.

## 2 Materials and methods

### 2.1 Intervention description: Cranky Uncle—Vaccine game

The text-based game uses cartoons, humor, and gamified features to teach players to recognize and resist vaccine misinformation. The “Cranky Uncle” character interacts with other characters to demonstrate logical fallacies and rhetorical techniques used to spread misinformation, such as “appeal to nature” or use of “fake experts,” while a health worker character provides factual vaccine information to correct Cranky Uncle. Players participate in quizzes to earn points, making Cranky Uncle angrier as they learn to recognize his tricks. The original version of the game—*Cranky Uncle*—was created in 2020 to combat climate change denialism (35, 36). The vaccine edition was later developed with UNICEF and additional project investigators from the Sabin Vaccine Institute and Irimi Company. The *Cranky Uncle – Vaccine* game has been adapted for different cultural contexts in Africa (34, 37–39). Evaluations using a pre-post design found that it statistically significantly improved myth discernment, vaccine attitudes, and intention to vaccinate in Uganda and Kenya (37), Ghana (39), and Rwanda (40), with these effects strongest among older participants and those with lower educational attainment (37). Using qualitative participatory research methods, the research team developed an Arabic-language version of the game for use among Arabic-speakers in Australia (41).

### 2.2 Cranky Uncle—Vaccine (Arabic) adaptation

**Figure 1:**
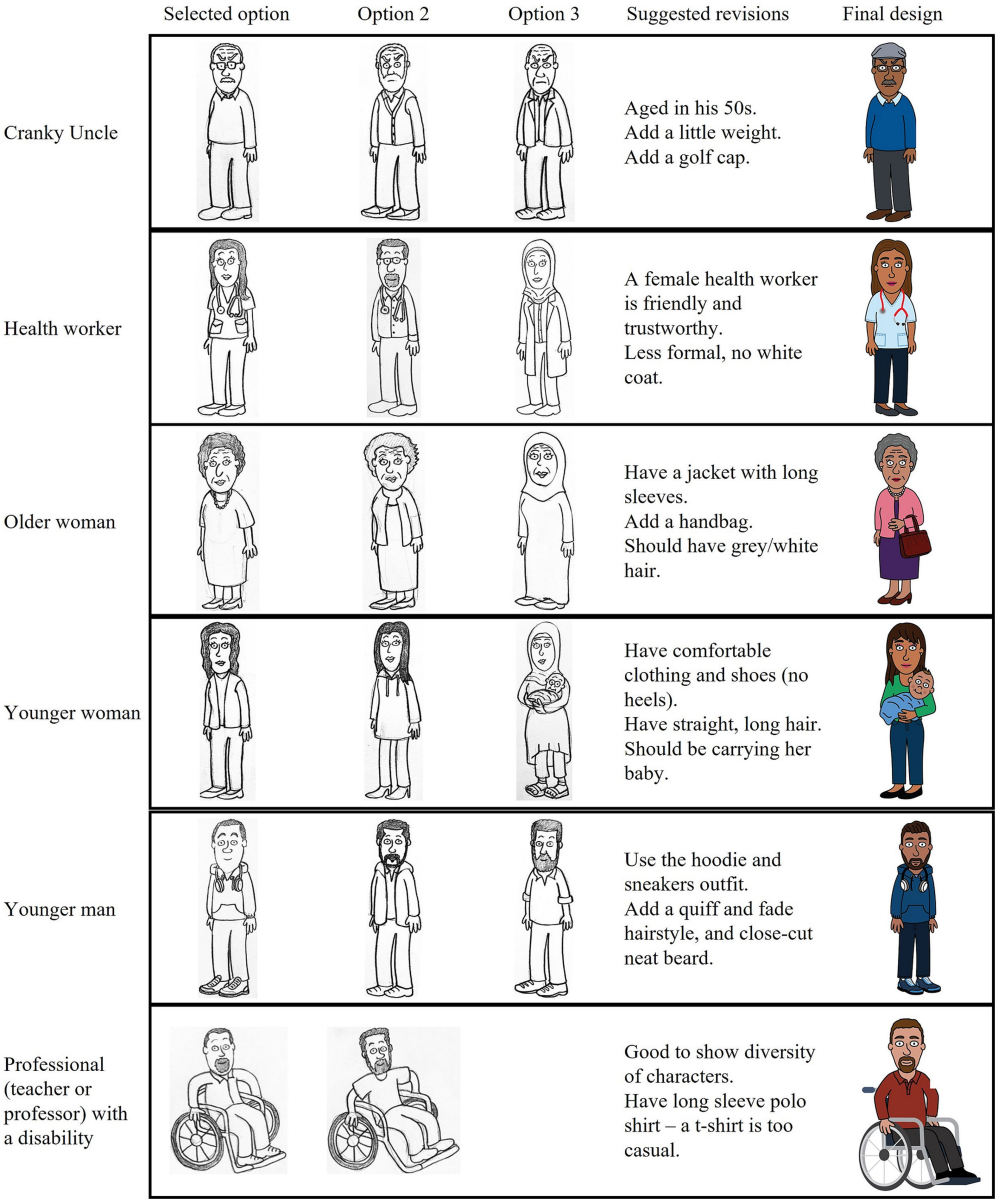
Initial sketches through to final design of Australian Cranky Uncle – Vaccine (Arabic) cartoon characters.

In a previous phase of the project (41), the research team worked with members of the Arabic-speaking community in Melbourne, Victoria to adapt the *Cranky Uncle – Vaccine* game’s characters and script. We followed the method previously used to successfully adapt the game in other countries (38). To adapt the characters, participants were presented with 3–5 images per character to facilitate discussions about suitable features and clothing. Participants noted that the characters must reflect the unique Arabic-Australian cultural identity of this community. Participants prioritized “comfortable” clothing such as low-heeled shoes and trousers. Cranky Uncle was given a cap and moustache because participants wanted him to be relatable, even if he is spreading misinformation. The research team proposed that the young woman wear a hijab to represent the people of Muslim faith in this linguistic community group. However, several male and female participants of Christian and Muslim faith stressed that it was inappropriate to “put a spotlight” on Muslims in this way. It was strongly preferred that their representation remain implicitly understood. This advice was also reinforced by the project’s Arabic-language community advisory group. The Modern Standard Arabic script was adapted to use simplified language, provide more information about vaccines, and refer to and refute misinformation commonly heard by participants. These adaptations were member checked with the community advisory group and additional minor changes were made (Figure 1). The game is available online (42).

### 2.3 Trial design and setting

This was a randomized controlled trial with a two-arm, parallel-group design and 1:1 participant randomization. The trial protocol was registered in the Australian and New Zealand Clinical Trials Registry (registration number: ACTRN12624000934549p). The trial took place online in the community, where participants completed trial activities in their chosen environment and time.

### 2.4 Participants and recruitment

Individuals were eligible if they were aged 16 years or older, could read Modern Standard Arabic, lived in Australia, and could independently play the game. Individuals were ineligible if they had previously participated in the focus group discussions or workshops conducted as part of this study (41).

Participants were recruited using convenience sampling via Arabic-language social networks, closed social media groups and snowball sampling. Recruitment was facilitated by the project’s Arabic-language community advisory group. Participant screening, consent, randomization and study data collection were managed using REDCap, a secure web-based software platform designed to support data capture for research studies (43, 44). Recruitment materials provided a link and QR code to the baseline REDCap survey’s landing page, which featured a brief description of the study, a downloadable participant information sheet, and informed consent form in Modern Standard Arabic. Contact details for the study team were available for any potential participants who wished to ask questions in either English or Arabic before providing consent. The project’s aim was presented as investigating ‘people’s attitudes to significant issues such as vaccines and climate change’ in order to blind participants to their group allocation.

### 2.5 Procedure

#### 2.5.1 Screening and baseline data collection

Participants completed screening questions to confirm they met the eligibility criteria described above after giving consent. Eligible participants proceeded to the baseline survey, where they answered demographic questions including gender, age, education level, years living in Australia, and postcode of residence, as well as the outcome measure items (vaccine attitudes, misinformation discernment, and institutional trust, described further below and in Supplementary materials).

#### 2.5.2 Randomization and blinding

At the end of the survey, they were instructed to click to proceed to an online activity, which triggered randomization. Participants were randomized in a 1:1 ratio to the intervention or control condition via an algorithm using permuted random blocks. A random number list was generated by a statistician not involved in the study. This list was uploaded to REDCap, which then automatically assigned enrolling participants to a group based on the next number in the list.

The intervention being tested was concealed from participants. The project’s aim was obfuscated in recruitment materials and surveys included items related to climate change that were not intended for nor used in analysis. Participants were not informed of their assignment (intervention vs. control) following their participation. Both activities were embedded into the REDCap survey architecture, permitting navigation between the surveys and intervention or control activities. Data were collected from October 2024 to January 2025.

#### 2.5.3 Intervention

Participants randomized to the intervention group were directed to the *Cranky Uncle - Vaccine (Arabic)* game in Modern Standard Arabic. Participation in the game was estimated to take 10–30 min. Participants who stopped playing the game mid-way received a reminder via email or text within 24 h, linking them back to the place where they stopped.

#### 2.5.4 Control

The research team was unable to find a similar freely available online educational game in the Arabic language. Therefore, an online educational video about climate change was selected as the control condition. We selected this topic because like vaccines, it is also the subject of misinformation (45, 46). We positioned the study as one about “facts and fiction in the internet age,” which allowed us to measure our required outcomes while retaining participant blinding to group allocation. The video was created and published on YouTube by the Intergovernmental Panel on Climate Change, lasted approximately 12 min, and was in English with Arabic subtitles (47). It contained no information about vaccines or infectious diseases. Participants allocated to the control group were directed to this video.

#### 2.5.5 Post-activity

Compliance with the study intervention was defined as completion of all levels of the game, and for the control arm, the video being watched in full. Compliance was established by the participant clicking the ‘next’ button, which did not appear until the game or video was complete, to progress to the post-activity survey, which repeated the baseline survey outcome measures. Participants who did not complete the survey within 24 h were sent a reminder with a direct link to it. At the end of the post-activity survey participants provided their first name and an email address and/or mobile phone number to receive an honorarium ($15). After 3 weeks a link to the follow-up survey was sent. Up to two reminders were sent to participants who did not complete the survey within 7 days of sending the initial link. Participants who completed the follow-up survey received a second honorarium ($5). The same set of outcome measure items were asked at baseline and both follow-ups.

### 2.6 Outcome measures

#### 2.6.1 Vaccine attitudes

Vaccine attitudes were assessed using the Vaccine Trust Indicator (VTI). The VTI is a short-form of a 120-item vaccination attitudes survey, containing six of those items (48). Items represent constructs associated with vaccine confidence, including trust in key vaccine stakeholders, vaccine knowledge and vaccine effectiveness and self-efficacy. Items are rated on an 11-point Likert scale, anchored at 0 = strongly disagree and 10 = strongly agree. The score is an unweighted average of the participants’ responses to each question and scaled to a 100-point scale. The mean and standard deviation for the general Australian population are M = 77.9, SD = 18 (48). While validity of the VTI has not been assessed specifically in an Arabic-speaking population, the instrument was translated, back translated to English to confirm translation accuracy, and reviewed by members of the community advisory group prior to use.

#### 2.6.2 Misinformation discernment

At the time of conducting this study no psychometrically validated scales of misinformation discernment existed (37), so misinformation discernment was measured using four vaccine fallacy and two vaccine fact items developed by the research team. The vaccine fallacy items were adapted from quiz questions in the *Cranky Uncle - Vaccine (Arabic)* game, and informed by common vaccine misinformation arguments in the scientific literature (37). The vaccine facts items were based on current evidence in the scientific literature. All items were rated on a 4-point scale, where 0 = mostly unbelievable, 1 = fairly unbelievable, 2 = fairly believable, and 3 = mostly believable. Fallacious items were reverse coded so that higher scores indicate more correct answers.

#### 2.6.3 Institutional trust

Institutional trust was measured using three of the nine items of the institutional trust subscale developed by Krastev et al. (49). The three items selected were conceptually and empirically most strongly associated with vaccine hesitancy, and that were not among the VTI items, i.e., trust in scientists, doctors and nurses, and the government. This scale was developed in accord with OECD guidelines for self-report measures of trust (20). While Krastev et al. (49) used a 5-point response scale, we used the OECD-recommended 11-point scale, anchored at 0 = strongly disagree and 10 = strongly agree, to reduce cognitive load for participants by presenting a consistent response format across two outcome measures. As with the VTI, the translated institutional trust instrument was reviewed for clarity and face validity by the community advisory group.

### 2.7 Analytical methods

The sample size was calculated using the VTI as the primary outcome measure. To detect a difference in means of 10 units in the VTI with 90% power, assuming a common standard deviation of 18 and using a two-sample t-test with a 5% two-sided significance level, a sample size of 70 in each group was required. This was increased to 100 per group, or 200 in total, to allow for an anticipated 30% loss to follow-up.

Demographic characteristics and baseline scale scores are presented by study group in the intention-to-treat (ITT) population. Categorical variables are reported as the number and percentage. Age, which had a skewed distribution, is summarized by the median, interquartile range (IQR) and range while baseline scale scores are summarized by the mean, standard deviation (SD) and range.

The analysis was conducted following the ITT principle. At both the immediate post-activity timepoint and the 3-week follow-up, greater than 10% of the primary (VTI) and secondary (institutional trust and misinformation discernment) outcome data were missing. Therefore, the main analysis was performed using multiple imputation at both timepoints. Imputations were generated using chained equations with 50 imputed datasets and 10 iterations between each imputation. Imputation was carried out separately by group to ensure that any treatment effects were maintained. Baseline characteristics, including baseline scores of key outcomes, as well as any variables that were predictors of missingness were included as auxiliary variables in the imputation model. Estimates of interest were obtained using Rubin’s rules (50).

We used linear regression to estimate between-group differences at the immediate post-activity timepoint, adjusting for baseline outcome scores. Missing outcome data were handled using multiple imputation. We used repeated measures linear regression to estimate between-group differences at the 3-week timepoint, also including the immediate post-activity timepoint, adjusting for baseline outcome scores and treatment by time interaction.

A sensitivity analysis was conducted running the main analysis models using available participant data only. Additional sensitivity analyses were conducted by adjusting for baseline VTI when the outcome was institutional trust and adjusting for baseline institutional trust when the outcome was VTI for all models analyzing the immediate post-activity timepoint. This was repeated for VTI and institutional trust scores at the 3-week follow-up. The analyses with the additional baseline covariates were conducted to show that the results were robust to the baseline covariates included.

A complete case subgroup analysis was conducted estimating the effect of treatment on the immediate and 3-week post-activity outcomes in subgroups according to the participants’ age (defined as age ≥36 and <36 years) and education (defined as up to Bachelor’s degree vs. Bachelor’s degree or post-graduate), adjusting for the baseline levels of the corresponding outcome. An interaction term between the subgroup and treatment group was included in each model.

The adjusted mean and 95% confidence interval (CI) in each group are presented. The adjusted mean difference is reported with its corresponding 95% CI and *p*-value.

A *post-hoc* analysis was performed on the items that make up each of the outcome measures. The mean difference was estimated using linear regression on each of the 11-point items for VTI and institutional trust at the immediate post-activity timepoints, adjusted for the corresponding baseline level. The adjusted mean difference and 95% CI are presented. The 4-point items that make up the misinformation discernment score were dichotomized to “correct belief” and “incorrect belief.” The risk difference was estimated using binomial regression on these binary variables, adjusted for the corresponding baseline level. The adjusted risk difference and 95% CI are presented graphically. This analysis was performed using available participant data only, analysing participants as randomized.

Analyses were performed using Stata version 18.0 (StataCorp, College Station, TX, USA,2023).

## 3 Results

### 3.1 Participants

**Figure 2:**
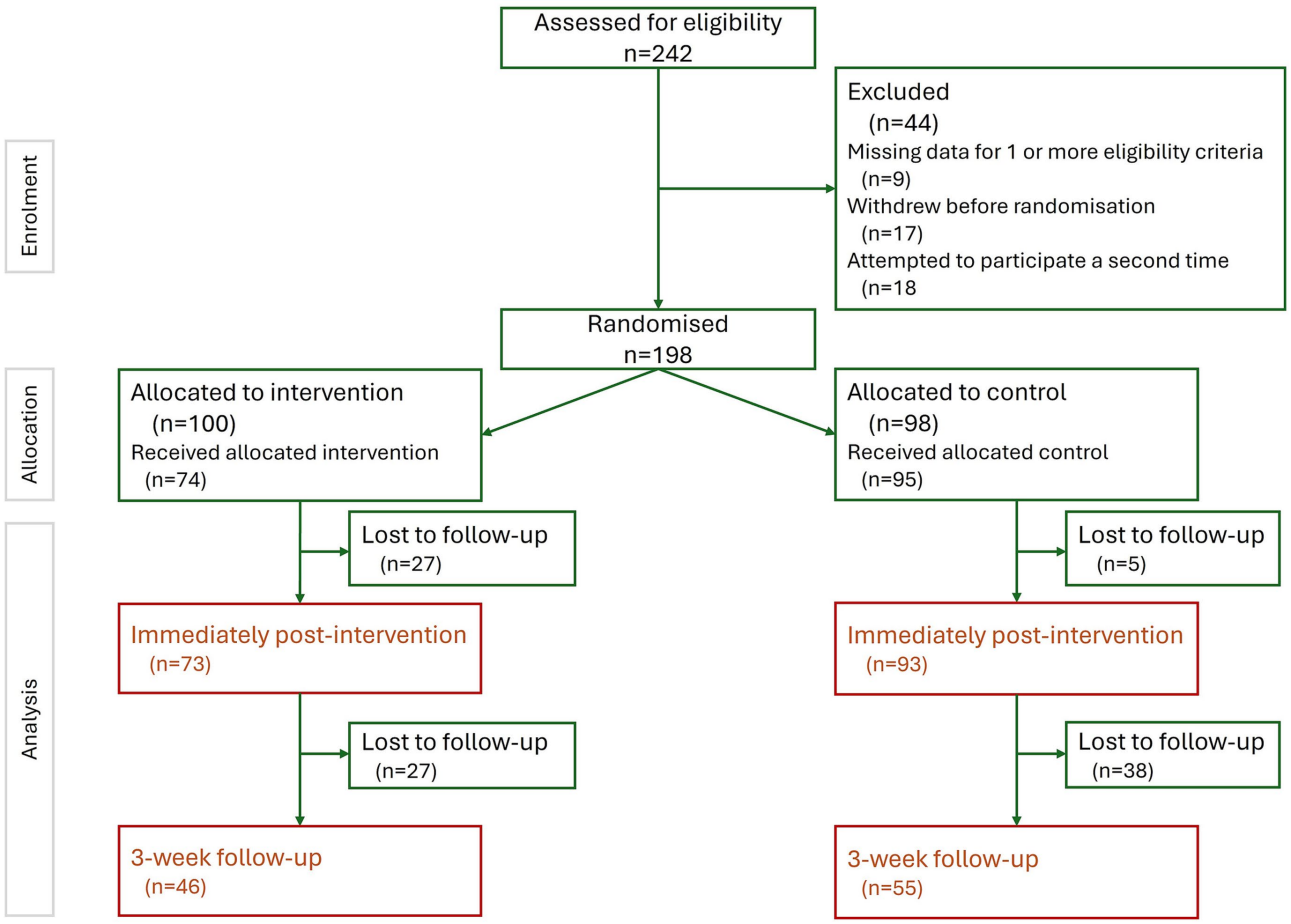
CONSORT diagram of the flow of participants throughout the trial.

Of 242 individuals who completed the screening survey, 44 were ineligible (see Figure 2 for ineligibility reasons). One hundred participants were randomized to the intervention and 98 to the control group. In the intervention group, 73 provided immediate post-intervention data and 46 provided data at the 3 week timepoint. In the control group, immediate post-intervention data were provided by 93 participants, with 55 completing the 3 week follow-up. Demographic characteristics were similar across the study groups, although slightly more men were allocated to the intervention group (54% vs. 45%) (Table 1). The baseline outcome measure scores were also similar across study groups. The mean (SD) VTI score for our sample was 61.8 (15.9), considerably lower than for the general Australian population (77.9) (18).

**Table 1:** Baseline demographic characteristics of study participants and scale scores.

Characteristics and scales	Randomized allocation		Total
	Intervention (game)	Control (video)	
	*n =* 100	*n =* 98	*n =* 198
Age (years), Median (IQR) [Range]	39 (32, 48) [16, 66]	37 (29, 47) [16, 71]	39 (31, 48) [16, 71]
Gender			
Woman	46 (46%)	53 (54%)	99 (50%)
Man	54 (54%)	44 (45%)	98 (49%)
Prefer not to say	0 (0%)	1 (1%)	1 (1%)
Highest level of education			
Less than a Bachelor degree	67 (67%)	59 (60%)	126 (64%)
Bachelor degree	33 (33%)	36 (37%)	69 (35%)
Post-graduate degree	0 (0%)	3 (3%)	3 (2%)
Years lived in Australia			
I was born here	1 (1%)	1 (1%)	2 (1%)
Less than 1 year	1 (1%)	1 (1%)	2 (1%)
1–3 years	21 (21%)	21 (21%)	42 (21%)
4–8 years	39 (39%)	37 (38%)	76 (38%)
More than 8 years	38 (38%)	38 (39%)	76 (38%)
Regionality			
Regional	1 (1%)	3 (3%)	4 (2%)
Major City	98 (98%)	91 (93%)	189 (95%)
Missing	1 (1%)	4 (4%)	5 (3%)
State of residence			
VIC	95 (95%)	96 (98%)	191 (96%)
NSW	4 (4%)	1 (1%)	5 (3%)
Missing	1 (1%)	1 (1%)	2 (1%)
Baseline scale scores			
VTI score [0–100], Mean (SD) Range	63.4 (17.2) [28–100]	60.2 (14.4) [25–100]	61.8 (15.9) [25–100]
Institutional trust score [0–30], Mean (SD) Range	18.2 (5.4) [10–30]	16.5 (4.8) [6–30]	17.4 (5.2) [6–30]
Misinformation discernment score [0–18], Mean (SD) Range	9.1 (2.5) [1–16]	8.7 (2.3) [3–14]	8.9 (2.4) [1–16]

### 3.2 Primary and secondary outcomes

The mean immediate post-activity VTI score (adjusted for baseline VTI score) in the intervention group was 67.9 (95% CI 65.3 to 70.5) and 67.7 (95% CI 65.2 to 70.1) in the control group, with a mean difference of 0.2 (95% CI −3.3 to 3.8) (Table 2). All other sensitivity analyses gave similar results. The multiple imputation (Table 2) and sensitivity analyses (Table 3) produced small adjusted mean differences across both primary and secondary outcomes. However, all 95% CIs include the possibility of no or clinically insignificant differences between study groups in vaccine attitudes, trust in institutions and accurate misinformation discernment immediately or 3 weeks after playing the game, after controlling for baseline scores.

**Table 2:** Vaccine Trust Indicator, institutional trust, and misinformation discernment, reporting adjusted mean scores by treatment group: adjusted mean difference with 95% CI using multiple imputation.

Outcome	Intervention (game) (*n =* 100)	Control (video) (*n =* 98)	Adjusted mean difference (95% CI)	*p*-value
	Adj. Mean (95% CI)	Adj. Mean (95% CI)		
Immediately post intervention				
Vaccine Trust Indicator^a^	67.9 (65.3, 70.5)	67.7 (65.2, 70.1)	0.2 (−3.3, 3.8)	0.89
Vaccine Trust Indicator^b^	68.7 (66.4, 71.0)	66.8 (64.7, 69.0)	1.9 (−1.3, 5.0)	0.24
Institutional trust^a^	18.8 (18.1, 19.4)	18.5 (17.8, 19.1)	0.3 (−0.6, 1.2)	0.49
Institutional trust^c^	18.8 (18.2, 19.4)	18.4 (17.9, 18.9)	0.4 (−0.4, 1.2)	0.29
Misinformation discernment^a^	10.2 (9.6, 10.7)	9.7 (9.2, 10.2)	0.5 (−0.3, 1.2)	0.23
3-week follow-up				
Vaccine Trust Indicator^d^	68.9 (64.9, 72.8)	71.7 (68.3, 75.1)	−2.8 (−8.0, 2.3)	0.28
Vaccine Trust Indicator^e^	69.5 (65.7, 73.3)	71.1 (67.7, 74.4)	−1.6 (−6.5, 3.4)	0.54
Institutional trust^d^	18.4 (17.4, 19.5)	18.8 (18.0, 19.7)	−0.4 (−1.8, 1.0)	0.59
Institutional trust^f^	18.5 (17.4, 19.5)	18.8 (17.9, 19.7)	−0.3 (−1.7, 1.1)	0.64
Misinformation discernment^d^	10.1 (9.4, 10.8)	9.8 (9.2, 10.5)	0.3 (−0.7, 1.3)	0.55

^a^Multiple imputation linear regression, adjusted for the baseline score. ^b^Multiple imputation linear regression adjusted for baseline score and baseline institutional trust score. ^c^Multiple imputation linear regression adjusted for baseline score and baseline VTI score. ^d^Multiple imputation repeated measures (mixed effects) linear regression, adjusted for the baseline score. ^e^Multiple imputation repeated measures (mixed effects) linear regression, adjusted for the baseline score and baseline institutional trust score. ^f^Multiple imputation repeated measures (mixed effects) linear regression adjusted for baseline score and baseline VTI score.

**Table 3:** Sensitivity analyses of the Vaccine Trust Indicator, institutional trust, and misinformation discernment, reporting adjusted mean scores by treatment group: adjusted mean difference with 95% CI.

Outcome	Intervention (game) (*n =* 100)		Control (video) (*n =* 98)			
	N*	Adj. mean (95% CI)	N*	Adj. mean (95% CI)	Adj. mean difference (95% CI)	*p*-value
Immediately post intervention: Adjusted for baseline score						
Vaccine Trust Indicator^a^	73	67.9 (65.2, 70.5)	93	66.5 (64.2, 68.9)	1.3 (−2.2, 4.9)	0.45
Vaccine Trust Indicator^b^	73	68.4 (66.0, 70.7)	93	66.1 (64.1, 68.2)	2.2 (−0.9, 5.4)	0.16
Institutional Trust^a^	73	18.3 (17.6, 19.0)	93	17.9 (17.3, 18.5)	0.4 (−0.5, 1.3)	0.39
Institutional trust^c^	73	18.3 (17.8, 18.9)	93	17.8 (17.3, 18.4)	0.5 (−0.3, 1.3)	0.20
Misinformation discernment^a^	70	10.3 (9.7, 10.8)	92	9.6 (9.1, 10.1)	0.6 (−0.1, 1.4)	0.10
3-week follow-up: Adjusted for baseline and immediate post intervention scores						
Vaccine Trust Indicator^d^	46	68.9 (65.4, 72.4)	55	71.3 (68.1, 74.4)	−2.3 (−7.0, 2.4)	0.33
Vaccine Trust Indicator^e^	46	69.1 (65.8, 72.4)	55	70.5 (67.5, 73.6)	−1.4 (−5.9, 3.1)	0.54
Institutional Trust ^d^	46	18.6 (17.7, 19.6)	55	19.0 (18.1, 19.8)	−0.3 (−1.6, 0.9)	0.59
Institutional trust^f^	46	18.6 (17.7, 19.5)	55	18.8 (18.0, 19.7)	−0.2 (−1.4, 1.0)	0.73
Misinformation discernment^d^	46	10.0 (9.3, 10.7)	55	10.0 (9.3, 10.6)	0.0 (−0.9, 1.0)	0.95

*N indicates available data included in the analysis at the specified time point. ^a^Linear regression, adjusted for the baseline score. ^b^Linear regression adjusted for baseline score and baseline institutional trust score. ^c^Linear regression adjusted for baseline score and baseline VTI score. ^d^Repeated measures (mixed effects) linear regression, adjusted for the baseline score. ^e^Repeated measures (mixed effects) linear regression, adjusted for the baseline score and baseline institutional trust score. ^f^Repeated measures (mixed effects) linear regression adjusted for baseline score and baseline VTI score.

At the 3-week follow-up, the mean differences for VTI and institutional trust were negative, indicating marginally higher sustained score improvement for the control group compared to the intervention group.

The subgroup analyses for age groups (36 + vs. <36 years) and education levels (less than Bachelor degree vs. Bachelor degree and above) showed no evidence of a difference in effectiveness between these groups across all outcomes and timepoints, with considerable overlap of the 95% CIs, and insignificant *p*-values for the interaction between treatment and subgroup (Supplementary materials).

### 3.3 *Post-hoc* item analyses

*Post-hoc* analyses were conducted to investigate differences between study groups immediately post-activity at the item level, and to compare with a previous evaluation of the Cranky Uncle game (38). Analysis was performed using available participant data only, i.e., without multiple imputation. At the immediate follow-up there were 73 and 93 participants in the intervention and control groups, respectively.

**Figure 3:**
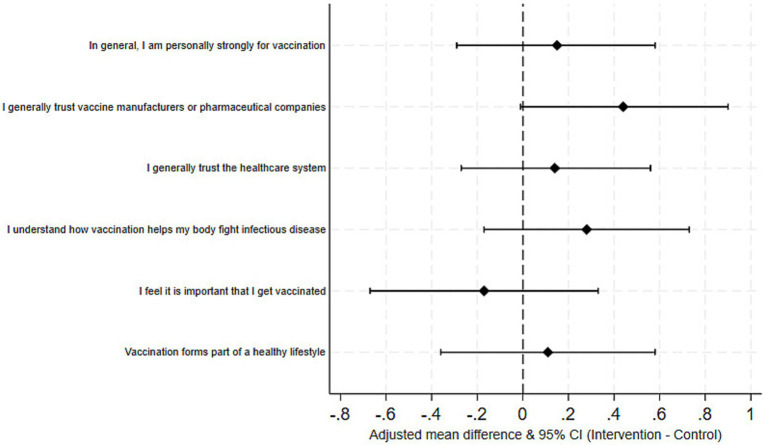
Forest plots of the adjusted mean difference and 95% CI of each item of the Vaccine Trust Indicator, immediate follow-up scores.

**Figure 4:**
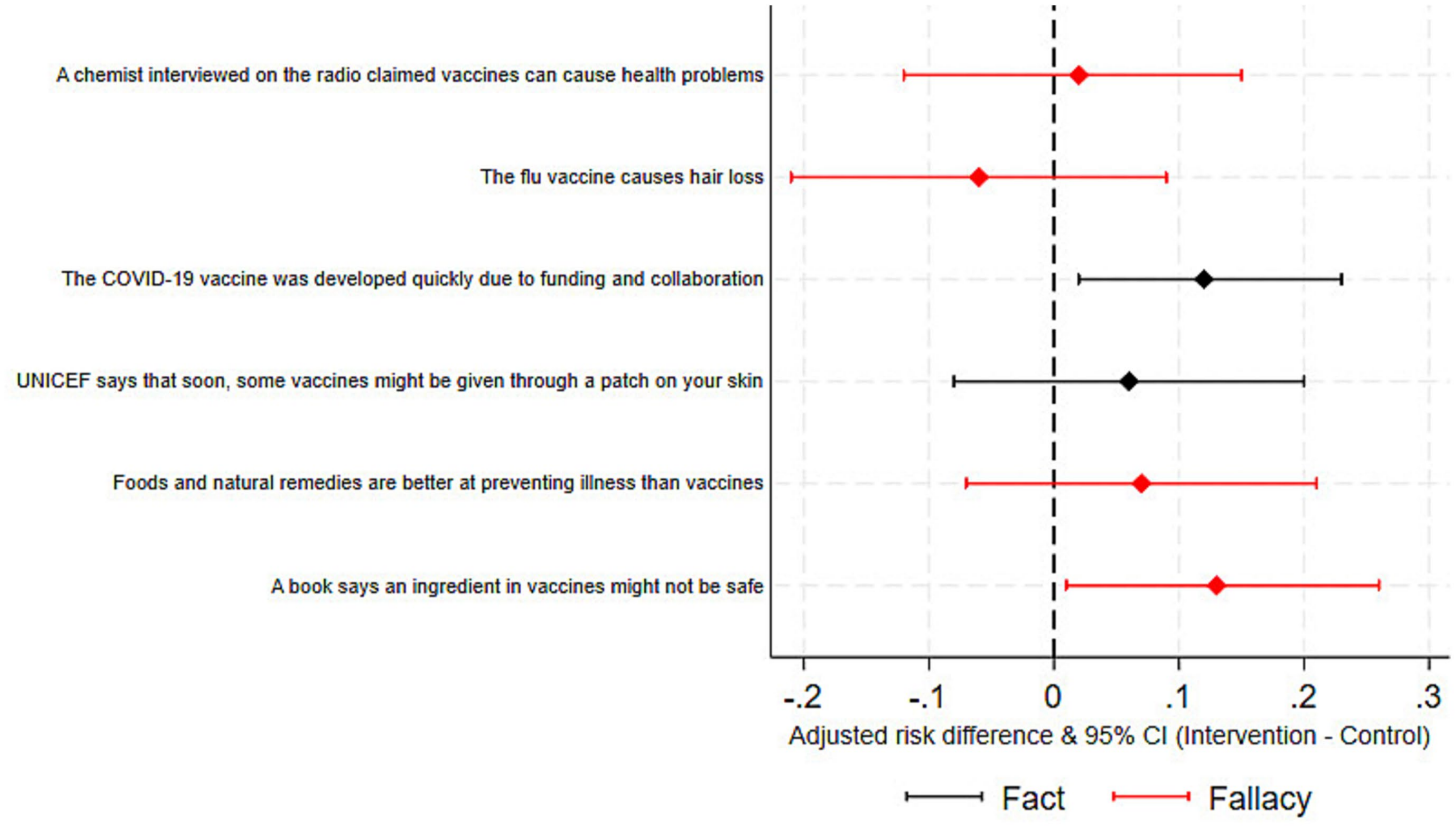
Forest plots of the adjusted mean difference and 95% CI of each item of the Misinformation Discernment Scale, immediate follow-up scores, correct belief.

**Figure 5:**
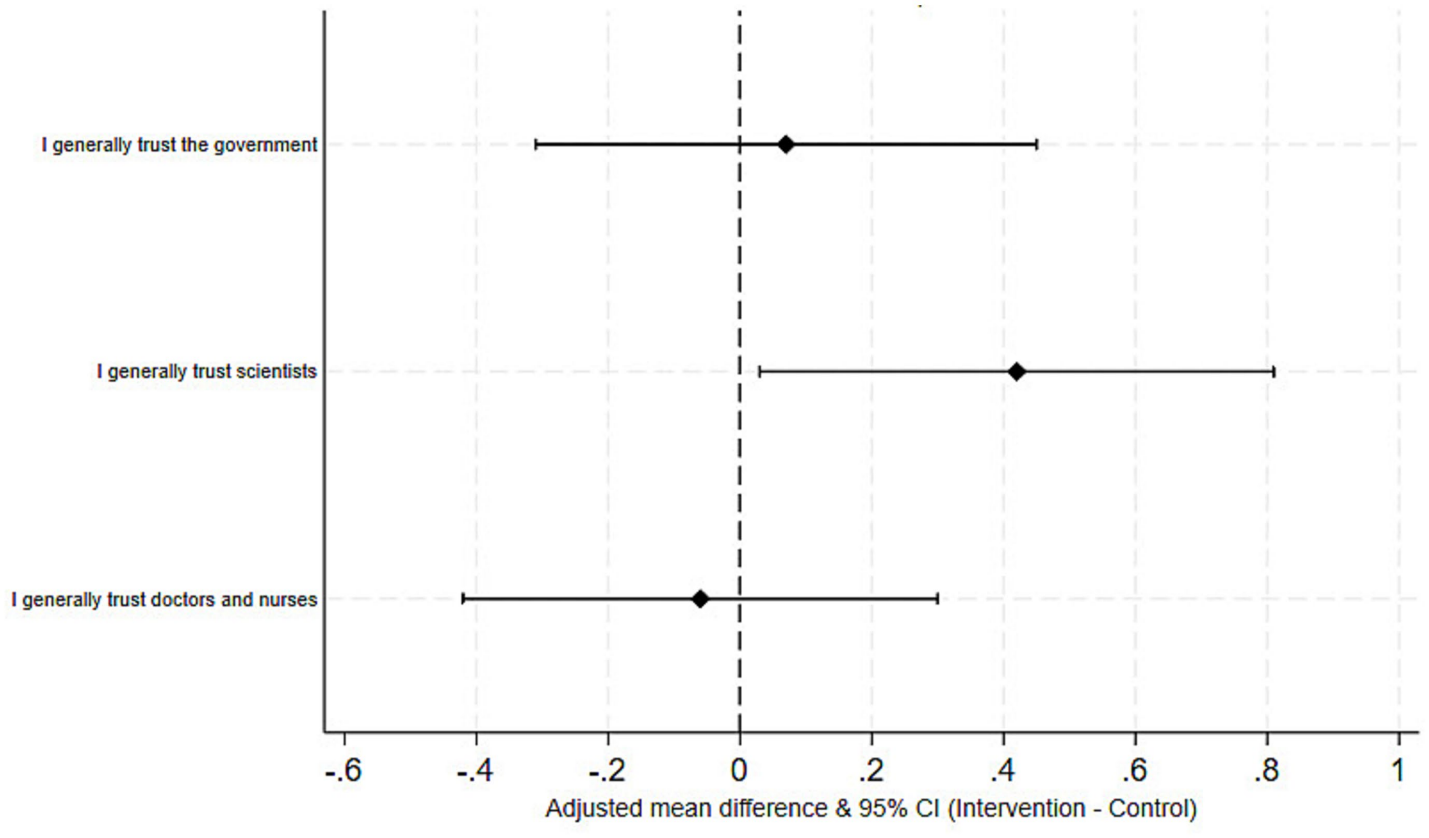
Forest plots of the adjusted mean difference and 95% CI of each item of the Institutional Trust Subscale, immediate follow-up.

Figures 3–5 present forest plots of the adjusted mean or risk difference and 95% CI for each item of the three outcome measures examined: VTI, institutional trust, and misinformation discernment. The VTI item with the largest difference in adjusted means was trust in manufacturers or pharmaceutical companies: 0.44. 95% CI (−0.01, 0.90) (Figure 3). Within the institutional trust scale, trust in scientists was higher in the intervention group: 0.42 95%CI (0.03, 0.81) (Figure 4). Among the misinformation discernment items, the intervention group had greater understanding that the COVID-19 vaccine was developed quickly due to funding and collaboration (adjusted risk difference 0.12 95%CI (0.02, 0.23)) and were more accurately able to detect the fallacy of ‘a book says an ingredient in vaccines might not be safe’ (adjusted risk difference 0.13 95% CI (0.01, 0.26)) (Figure 5).

## 4 Discussion

This randomized controlled trial examined the effectiveness of the *Cranky Uncle – Vaccine (Arabic)* game in improving vaccine attitudes, institutional trust and misinformation discernment compared to watching a control video on climate change, but no clinically significant difference between the interventions was found.

Our findings contrast with those of many other evaluations of psychological inoculation interventions, which report positive results (27–29, 31, 33, 51, 52), though there is substantial heterogeneity of study designs and outcome measures (29, 53). For example, a meta-analysis (29) of 15 studies about psychological inoculation of health misinformation with 6,732 subjects found that psychological inoculation effectively reduced the perceived credibility of misinformation and intentions to share it. However, it did not affect perceived credibility of correct information, nor intentions to share it. Similarly, a pre-post evaluation of the *Cranky Uncle – Vaccine* game adapted for Uganda and Kenya found modest but statistically significant improvements in attitudes and misinformation discernment after gameplay (37). That study also found that older participants showed significantly greater improvement after playing the game compared with younger participants, whereas our study found no age or educational level influence (37).

Comparison with past studies is further complicated by the fact that this emergent field is still developing standardized outcome measures and intervention conditions (54). Like us, many researchers develop their own misinformation discernment scales (55, 56). While this is a recognized limitation, developing an instrument that can evaluate interventions on a variety of topics is a challenge. For instance, the recently-validated Misinformation Susceptibility Test (MIST) instrument uses standardized news headlines to assess misinformation discernment, which is not sufficiently relevant to the *Cranky Uncle – Vaccine (Arabic)* intervention (53). Psychological inoculation research spans a wide range of domains, from political misinformation and climate denialism to health misinformation games (28, 29, 57, 58), and experts are increasingly calling for coordinated efforts to address methodological limitations (54).

We examined each item separately to identify differences between the control and intervention scores that might have been obscured by using them in a summed scale. The scores indicated a slightly greater improvement in the intervention group across most attitudinal items, with greatest improvement in trust in scientists and pharmaceutical companies. The misinformation discernment items that showed the largest difference between groups at the immediate timepoint were a fallacy (“A book says an ingredient in vaccines might not be safe. The author argues that if a vaccine is not safe for all people, then it should not be given”) and a fact (“The COVID-19 vaccine was developed quickly because so many countries shared their scientists, research data, and money”) (see Figure 4). However, all values in the 95% CIs are less than one, which for the 11-point items may indicate a difference that is not clinically meaningful. Interestingly, there is mixed evidence that psychological inoculations promote generalized skepticism of all information – both facts and fallacies – and mistrust in institutions (29, 31, 59). As noted earlier and by others (29, 56, 60), this evidence is inconsistent across studies and may be better explained by the need for standardized measures than by an empirically reliable effect.

Vaccine attitudes and institutional trust have long been recognized as key determinants of vaccination intentions and uptake (23, 61, 62). However, we have been unable find an established, empirically derived theoretical framework linking misinformation beliefs with these factors. Our findings suggest that while misinformation discernment may influence upstream factors like institutional trust, it may function as a relatively distal predictor of downstream behavioral outcomes such as vaccine intentions and uptake. Future research should examine the direct and interaction effects between trust, attitudes, and misinformation discernment to advance this field. Early stage inroads have been made in theoretical framework and scale development [e.g., (63)], however further research should explore the strength and directionality of the relationship between this form of trust and vaccine uptake behavior, as this could have meaningful implications for public health communication strategies. Bridging this gap in public health interventions may require supplementary interventions that address affective, social and environmental drivers of vaccine attitudes in conjunction with cognitive ones (64, 65).

As well as being successfully adapted for the Arabic-speaking community in Australia, a specific cultural-linguistic subgroup affected by vaccine misinformation (13), the *Cranky Uncle – Vaccine* game has also been adapted for several East African countries (38). This demonstrates the versatility of the game for a global audience, and its utility to global and local health promotion organizations. However, this study also highlighted the importance of using the appropriate dissemination method for each community. We heard from the community and the project’s advisory group that the game would be well received if disseminated in structured educational group settings. This may also have been a more effective trial setting, and one that should be explored in future research.

### 4.1 Limitations

This study has several potential limitations. Twenty-six participants allocated to the intervention group dropped out before or while playing the game, compared to just three participants after allocation to the control group. This rate of drop-out may limit the reliability and generalisability of findings. Unfortunately, the reasons for this drop-out rate cannot be identified because the intervention was administered by participants without researcher oversight. Due to a lack of appropriate measurement instruments validated for use with Arabic-speakers, we had to adapt or develop our own instruments which may have introduced bias. Furthermore, there remains limited evidence from which to assess clinical significance of findings achieved from a psychological inoculation intervention (29, 33, 37). The control condition, a climate change video in English with Arabic subtitles, was passive rather than active and may not have been directly comparable to the intervention. Feasibility constraints related to Arabic-language, online media, project resources and concerns about excessive burden to participants limited the type of control condition that could be administered. Finally, we were unable to validate our misinformation items via cognitive and psychometric testing due to feasibility constraints. Future research should aim to develop psychometrically robust measures for use in psychological inoculation research, including in diverse languages.

### 4.2 Future research

This study is the latest to demonstrate the adaptability of the *Cranky Uncle – Vaccine (Arabic)* game to different cultural and linguistic groups. The community workshop process worked well to identify the necessary adaptations to characters and script to meet community needs and expectations.

As an emergent field, each study of a psychological inoculation intervention tends to raise more questions than it answers. There are currently robust discussions about definitions, methodologies, data analysis approaches, and implementation and outcome measures (54, 66, 67). For example, an insightful yet parsimonious theoretical framework proposed by Zmigrod and colleagues (68) advances theoretical understanding and suggests more work to be done in relation to psychometrically robust outcome measures for intervention trials.

## 5 Conclusion

This randomized controlled trial examined the effectiveness of the *Cranky Uncle – Vaccine (Arabic)* game in improving vaccine attitudes, misinformation discernment and institutional trust. The findings showed small and non-significant effects on the outcome measures. The study has highlighted the gaps in this emergent field of research, including the need for standardized measures developed through cognitive testing and psychometric validation. Additionally, the global utility of such games requires culturally appropriate translations of measures. Similarly, further theoretical development is also essential, particularly in building predictive models of vaccine behavior that consider factors like e-health literacy and other relevant covariates. Future implementation trials should test this or other misinformation interventions in structured educational group settings to support broader application, impact and sustainability.

## Data Availability

The raw data supporting the conclusions of this article will be made available by the authors, without undue reservation.
