# Supplementary material for: Effectiveness of an online vaccine misinformation game for Arabic-speaking Australians: a randomized controlled trial
Source: Front Public Health. 2026 May 28;14:1768612. doi: 10.3389/fpubh.2026.1768612 (PMC13254167; doi:10.3389/fpubh.2026.1768612)
Supplement: Supplementary file 1 [file Table_1.docx]

**SUPPLEMENTAL MATERIAL**

**Survey instrument (repeated at baseline, immediately post-activity, and 3 weeks post-activity).** Climate items were included to blind participants to their group allocation but were not analysed.

**YOUR VIEWS ABOUT SOCIAL ISSUES**

**Please rate the following statements out of 10:** *[Randomized items]*

|  | *Measure* | *Item* | *Responses* | |
| --- | --- | --- | --- | --- |
| *1* | *VTI* | Thinking about **vaccination** in general, would you say you are personally… | 0=Strongly against it | 10=Strongly for it |
| *2* | *Climate attitudes (for blinding)* | Thinking about **renewable energy** in general, would you say you are personally…. | 0=Strongly against it | 10=Strongly for it |

**Please rate how strongly you agree with the following statements, with 0 being Strongly Disagree and 10 being Strongly Agree:** *[Randomized items]*

|  | *Measure* | *Item* | *Responses* | |
| --- | --- | --- | --- | --- |
| *4* | *VTI* | I generally trust vaccine manufacturers or pharmaceutical companies | 0=Strongly Disagree | 10=Strongly Agree |
| *5* | *VTI* | I generally trust the healthcare system | 0=Strongly Disagree | 10=Strongly Agree |
| *6* | *Climate attitudes (for blinding)* | I generally trust the renewable energy industry | 0=Strongly Disagree | 10=Strongly Agree |
| *7* | *VTI* | I understand how vaccination helps my body fight infectious disease | 0=Strongly Disagree | 10=Strongly Agree |
| *8* | *VTI* | I feel it is important that I get vaccinated | 0=Strongly Disagree | 10=Strongly Agree |
| *9* | *VTI* | Vaccination forms part of a healthy lifestyle | 0=Strongly Disagree | 10=Strongly Agree |
| *10* | *Climate attitudes (for blinding)* | Addressing climate change is important for the future | 0=Strongly Disagree | 10=Strongly Agree |
| *11* | *IT* | I generally trust the government | 0=Strongly Disagree | 10=Strongly Agree |
| *12* | *IT* | I generally trust scientists | 0=Strongly Disagree | 10=Strongly Agree |
| *13* | *IT* | I generally trust doctors and nurses | 0=Strongly Disagree | 10=Strongly Agree |

*[VTI: Vaccine Trust Index (Ellingson, 2023)*

*IT: Institutional Trust subscale (Krastev et al., 2023)]*

**FACTS AND FICTION** *[Randomized items]*

**Please read these statements carefully and answer whether you think the statement is mostly unbelievable, fairly unbelievable, fairly believable, mostly believable.**

|  | *Statement* | *Response set(coding)* | *Coding* | *Topic* |
| --- | --- | --- | --- | --- |
| *1* | A famous radio host interviewed a chemist who said that vaccines can cause health problems. | - Mostly unbelievable(3), - Fairly unbelievable(2), - Fairly believable(1), - Mostly believable(0) | *Reverse coded (fallacy)* | *Vaccines* |
| *2* | My hair is thinner ever since I got the flu shot. The flu vaccine must cause hair loss, but doctors don’t seem to care. | - Mostly unbelievable(3), - Fairly unbelievable(2), - Fairly believable(1), - Mostly believable(0) | *Reverse coded (fallacy)* | *Vaccines* |
| *3* | UNICEF says that soon, some vaccines might be given through a patch on your skin instead of an injection. | - Mostly unbelievable(0), - Fairly unbelievable(1), - Fairly believable(2), - Mostly believable(3) | *N/A (fact)* | *Vaccines* |
| *4* | The COVID-19 vaccine was developed quickly because so many countries shared their scientists, research data, and money. | - Mostly unbelievable(0), - Fairly unbelievable(1), - Fairly believable(2), - Mostly believable(3) | *N/A (fact)* | *Vaccines* |
| *5* | The companies that make solar panels are pushing the idea of "climate change" to make electricity more expensive. | - Mostly unbelievable(3), - Fairly unbelievable(2), - Fairly believable(1), - Mostly believable(0) | *Reverse coded (fallacy)* | *Climate (for blinding)* |
| *6* | Climate scientists are making climate science out to be much worse than it is because otherwise they would have nothing to research and be out of a job. | - Mostly unbelievable(3), - Fairly unbelievable(2), - Fairly believable(1), - Mostly believable(0) | *Reverse coded (fallacy)* | *Climate (for blinding)* |
| *7* | Over 97% of climate scientists agree that humans are causing global warming. | - Mostly unbelievable(0), - Fairly unbelievable(1), - Fairly believable(2), - Mostly believable(3) | *N/A (fact)* | *Climate (for blinding)* |
| *8* | Fast-flowing cold rivers are warmer now and this has caused a big drop in salmon populations in those rivers. Climate change caused this. | - Mostly unbelievable(0), - Fairly unbelievable(1), - Fairly believable(2), - Mostly believable(3) | *N/A (fact)* | *Climate (for blinding)* |
| *9* | The foods and natural remedies that our grandmothers gave us are better at preventing illness than vaccines. | - Mostly unbelievable(3), - Fairly unbelievable(2), - Fairly believable(1), - Mostly believable(0) | *Reverse coded (fallacy)* | *Vaccines* |
| *10* | A book says an ingredient in vaccines might not be safe. The author argues that if a vaccine isn’t safe for all people, then it shouldn’t be given. | - Mostly unbelievable(3), - Fairly unbelievable(2), - Fairly believable(1), - Mostly believable(0) | *Reverse coded (fallacy)* | *Vaccines* |

**Subgroup analyses results:** Adjusted mean scores by age and education measured at randomization: adjusted mean difference with 95% CI

**Supplemental Table 1**. Immediate follow-up, VTI

|  | **Intervention (game)**  **Adjusted Mean ( 95% CI)**  **(n=73)** | **Control**  **Adjusted Mean (95% CI)**  **(n=93)** | **Adjusted Mean Difference (95% CI)** | **p-value** |
| --- | --- | --- | --- | --- |
| **Primary analysis (ITT)** |  |  |  |  |
| **Age** |  |  |  | 0.16 |
| ≥ 36 years | 67.9 (64.6, 71.1) | 64.3 (61.2, 67.4) | 3.6 (-0.9, 8.1) |  |
| < 36 years | 67.9 (63.4, 72.4) | 69.4 (65.9, 72.9) | -1.5 (-7.2, 4.1) |  |
|  |  |  |  |  |
| **Education** |  |  |  | 0.13 |
| Less than a Bachelor degree | 64.4 (61.4, 67.5) | 64.5 (61.6, 67.4) | -0.1 (-4.2, 4.1) |  |
| Bachelor and above | 74.9 (70.6, 79.3) | 69.6 (66.1, 73.1) | 5.4 (-0.2, 11.0) |  |

**Supplemental Table 2.** Immediate follow-up, institutional trust

|  | **Intervention (game)**  **Adjusted Mean ( 95% CI)**  **(n=73)** | **Control**  **Adjusted Mean (95% CI)**  **(n=93)** | **Adjusted Mean Difference (95% CI)** | **p-value** |
| --- | --- | --- | --- | --- |
| **Primary analysis (ITT)** |  |  |  |  |
| **Age** |  |  |  | 0.92 |
| ≥ 36 years | 18.0 (17.2, 18.8) | 17.6 (16.8, 18.4) | 0.4 (-0.7, 1.6) |  |
| < 36 years | 18.8 (17.7, 20.0) | 18.3 (17.4, 19.2) | 0.5 (-0.9, 2.0) |  |
|  |  |  |  |  |
| **Education** |  |  |  | 0.92 |
| Less than a Bachelor degree | 17.8 (17.0, 18.6) | 17.4 (16.6, 18.1) | 0.4 (-0.7, 1.5) |  |
| Bachelor and above | 19.2 (18.1, 20.4) | 18.7 (17.8, 19.6) | 0.5 (-0.9, 2.0) |  |

**Supplemental Table 3**. Immediate follow-up, misinformation discernment

|  | **Intervention (game)**  **Adjusted Mean ( 95% CI)**  **(n=70)** | **Control**  **Adjusted Mean (95% CI)**  **(n=92)** | **Adjusted Mean Difference (95% CI)** | **p-value** |
| --- | --- | --- | --- | --- |
| **Primary analysis (ITT)** |  |  |  |  |
| **Age** |  |  |  | 0.92 |
| ≥ 36 years | 10.1 (9.4, 10.8) | 9.4 (8.8, 10.1) | 0.7 (-0.3, 1.6) |  |
| < 36 years | 10.7 (9.7, 11.7) | 9.9 (9.2, 10.7) | 0.7 (-0.5, 2.0) |  |
|  |  |  |  |  |
| **Education** |  |  |  | 0.39 |
| Less than a Bachelor degree | 9.5 (8.9, 10.2) | 9.0 (8.4, 9.6) | 0.6 (-0.3, 1.4) |  |
| Bachelor and above | 11.8 (10.9, 12.8) | 10.7 (9.9, 11.4) | 1.2 (0.0, 2.4) |  |

**Supplemental Table 4**. 3 week follow-up, VTI

|  | **Intervention (game)**  **Adjusted Mean ( 95% CI)**  **(n=10073** | **Control**  **Adjusted Mean (95% CI)**  **(n=93)** | **Adjusted Mean Difference (95% CI)** | **p-value** |
| --- | --- | --- | --- | --- |
| **Primary analysis (ITT)** |  |  |  |  |
| **Age** |  |  |  | 0.63 |
| ≥ 36 years | 69.8 (65.7, 73.8) | 69.4 (65.3, 73.6) | 0.3 (-5.5, 6.1) |  |
| < 36 years | 66.5 (59.8, 73.2) | 73.8 (68.9, 78.6) | -7.3 (-15.6, 1.0) |  |
|  |  |  |  |  |
| **Education** |  |  |  | 0.72 |
| Less than a Bachelor degree | 66.4 (62.4, 70.4) | 69.3 (65.4, 73.3) | -2.9 (-8.6, 2.7) |  |
| Bachelor and above | 75.0 (68.7, 81.3) | 74.6 (69.7, 79.4) | 0.4 (-7.5, 8.4) |  |

**Supplemental Table 5**. 3 week follow-up, institutional trust

|  | **Intervention (game)**  **Adjusted Mean ( 95% CI)**  **(n=73)** | **Control**  **Adjusted Mean (95% CI)**  **(n=93)** | **Adjusted Mean Difference (95% CI)** | **p-value** |
| --- | --- | --- | --- | --- |
| **Primary analysis (ITT)** |  |  |  |  |
| **Age** |  |  |  | 0.99 |
| ≥ 36 years | 18.8 (17.7, 19.9) | 19.4 (18.2, 20.5) | -0.6 (-2.1, 1.0) |  |
| < 36 years | 18.1 (16.3, 19.9) | 18.4 (17.1, 19.7) | -0.3 (-2.6. 2.0) |  |
|  |  |  |  |  |
| **Education** |  |  |  | 0.38 |
| Less than a Bachelor degree | 18.7 (17.6, 19.8) | 18.6 (17.5, 19.7) | 0.1 (-1.4, 1.7) |  |
| Bachelor and above | 18.4 (16.7, 20.2) | 19.6 (18.3, 21.0) | -1.2 (-3.4, 1.0) |  |

**Supplemental Table 6**. 3 week follow-up, misinformation discernment

|  | **Intervention (game)**  **Adjusted Mean ( 95% CI)**  **(n=71)** | **Control**  **Adjusted Mean (95% CI)**  **(n=92)** | **Adjusted Mean Difference (95% CI)** | **p-value** |
| --- | --- | --- | --- | --- |
| **Primary analysis (ITT)** |  |  |  |  |
| **Age** |  |  |  | 0.10 |
| ≥ 36 years | 10.5 (9.6, 11.3) | 10.0 (9.1, 10.8) | 0.5 (-0.7, 1.7) |  |
| < 36 years | 8.6 (7.2, 9.9) | 9.9 (8.9, 10.9) | -1.3 (-3.0, 0.4) |  |
|  |  |  |  |  |
| **Education** |  |  |  | 0.88 |
| Less than a Bachelor degree | 9.7 (8.9, 10.5) | 10.0 (9.2, 10.8) | -0.2 (-1.4, 0.9) |  |
| Bachelor and above | 10.5 (9.2, 11.8) | 9.9 (8.9, 10.9) | 0.6 (-1.0, 2.2) |  |
